# Impact of frailty on infection risk in non-transplant eligible multiple myeloma patients: a systematic review and meta-analysis

**DOI:** 10.1038/s41375-026-02880-y

**Published:** 2026-02-17

**Authors:** Federico Spataro, Giuseppe Armentaro, Giuseppe Di Gioia, Pierluigi Meloni, Ilaria Rossi, Michela Williams, Giulio Francesco Romiti, Rosa Talerico, Rosanna Villani, Vanessa Desantis, Hermann Einsele, Leonardo Bencivenga, Antonio Giovanni Solimando

**Affiliations:** 1 Department of Precision and Regenerative Medicine and Ionian Area - DiMePRe-J, Section of Pharmacology, University of Bari Aldo Moro, Bari, Italy; 2 Geriatrics Division, “Renato Dulbecco” University Hospital of Catanzaro, Catanzaro, Italy; 3 Department of Medical and Surgical Sciences, University of Foggia, Foggia, Italy; 4 Department of Medical, Surgical and Experimental Sciences, University of Sassari, Sassari, Italy; 5 Department of Medicine and Aging Science, “Clinica Medica” Institute, ‘SS Annunziata’ Hospital, “G. d’Annunzio” University, Chieti, Italy; 6 Center for Basic and Clinical Immunology Research (CISI), WAO Center of Excellence, University of Naples Federico II, Naples, Italy; 7 Department of Wellbeing, Health and Environmental Sustainability, Sapienza University of Rome, Rome, Italy; 8 Department of Geriatric, Orthopedic, and Rheumatologic Sciences, Fondazione Policlinico Universitario A. Gemelli IRCCS, Università Cattolica del Sacro Cuore, Rome, Italy; 9 Liver Unit, Department of Medical and Surgical Sciences, University of Foggia, Foggia, Italy; 10 Department of Internal Medicine II, University Hospital of Würzburg, Würzburg, Germany; 11 Department of Translational Medical Sciences, University of Naples “Federico II”, Naples, Italy; 12 Department of Precision and Regenerative Medicine and Ionian Area - DiMePRe-J, Guido Baccelli Unit of Internal Medicine, School of Medicine, University of Bari Aldo Moro, Bari, Italy

**Keywords:** Risk factors, Disease prevention

## To the Editor

Multiple myeloma (MM) is a hematologic malignancy characterized by clonal plasma cell proliferation in the bone marrow, resulting in immune suppression, bone destruction, and end-organ damage. It predominantly affects older adults, with a median diagnosis age of 66–70 years [1]. With the aging population, many patients are ineligible for high-dose chemotherapy with autologous stem cell transplantation (ASCT) due to comorbidities or frailty, making frailty assessment essential in treatment planning.

Frailty in MM is evaluated using the International Myeloma Working Group Frailty Index (IMWG-FI) and the Simplified Frailty Score [2, 3]. The IMWG-FI classifies patients as fit, intermediate, or frail based on age, comorbidities, and functional status, guiding treatment intensity due to the higher risk of adverse events in frail patients [2]. The Simplified Frailty Score uses provides a quicker assessment using age, performance status, and comorbidities [3].

Infections, especially grade 3–4, are major MM complications, leading to longer hospitalization and higher mortality [4]. Susceptibility results from immune dysfunction, bone marrow suppression, and treatment-related immunosuppression. Although predictive models such as FIRST, GEM-PETHEMA, and IRMM scores estimate infection risk using clinical and laboratory data, none integrate frailty classification in MM.

Given the growing emphasis on frailty assessments in MM clinical trials, standardizing frailty definitions and their impact on infection risk and overall patient outcomes has become a priority.

To address this gap, we performed a systematic review and meta-analysis, according to the Preferred Reporting Items for Systematic Reviews and Meta-Analyses (PRISMA) guidelines (PROSPERO registration ID: CRD420250654904), to evaluate the influence of frailty on the risk of severe infections (grade 3–4) in newly diagnosed MM (NDMM) patients ineligible for ASCT, by comparing infection rates among fit, intermediate, and frail individuals [5]. We conducted a comprehensive search of the MEDLINE and LILACS databases from inception (no backwards time limit) to February 1st, 2025.

The primary outcome was the risk of infection, calculated as the proportion of patients with grade 3–4 infections within each frailty category, expressed as risk ratios (RR) and pooled using the DerSimonian and Laird random-effects model, comparing non-frail (fit plus intermediate) vs frail patients [6, 7]. Subgroup analyses further compared infection risk across the different frailty categories. Heterogeneity was assessed using the χ² test and *I*² statistic.

Evidence certainty was graded by the GRADE approach [8]. To assess study quality, we applied the Quality Appraisal of Case Series Studies Checklist developed by the Institute of Health Economics (IHE) (accessible at http://www.ihe.ca/research-programs/rmd/cssqac/cssqac-about). Full methods are provided in the Supporting Information.

Five articles met the inclusion criteria and were included in the meta-analysis (Supplementary Fig. 1 and Supplementary Table 1). The overall certainty of the evidence for the risk of infection outcome was judged to be high (Supplementary Table 2). Supplementary Table 3 summarizes the five studies. Only one study had a retrospective design. Two trials stratified results by treatment regimen: Mateos et al. [9] compared patients receiving daratumumab, bortezomib, melphalan, and prednisone (DVMP) with those given bortezomib, melphalan, and prednisone (VMP), while Facon et al. [10] compared daratumumab, lenalidomide, and dexamethasone (DRd) with lenalidomide and dexamethasone (Rd). Stege et al. [11] (frail patients) and Groen et al. [12] (intermediate patients) both reported data from the HOVON-143 trial; to avoid duplication and allow appropriate non-frail *vs* frail comparison, these were treated as a single study (“Stege-Groen”) in the forest plot.

To assess frailty, Mateos et al. [9] and Facon et al. [10] studies used the Simplified Frailty Scale, Stege-Groen utilized the IMWG-FI, and Zhang et al. [13] employed the DynaFiT.

The baseline patient population included 1663 individuals (806 females, 48.5%), with a mean age of 73.6 years, of whom 1132 completed the studies. The mean duration of treatment was 27.2 months. A total of 434 patients (26.1%) developed infections.

**Fig. 1:**
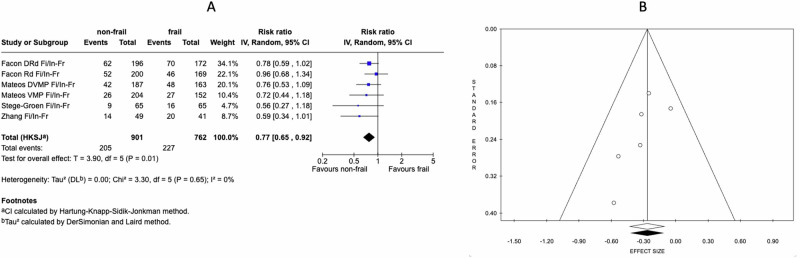
Meta-analysis and funnel plot for non-frail versus frail patients. Fi, fit; Fr, frail; In, intermediate; Fi/In, sum of fit and intermediate patients representing the non-frail group; RR, risk ratio. **A** Meta-analysis on non-frail vs frail multiple myeloma patient assessing the risk of infection, reported as RR. **B** Funnel plot under random effect model.

Compared to frail patients, non-frail (fit plus intermediate) ones showed a RR of 0.77 (95% CI: 0.65–0.92), indicating a 23% lower risk of infection in non-frail patients with heterogeneity (*I*^2^) of 0% (Fig. 1). A sensitivity analysis, including only prospective studies, excluding the single retrospective study, was also performed. This pooled analysis yielded a RR of 0.71 (95% CI: 0.48–1.07) (Supplementary Fig. 2).

**Fig. 2:**
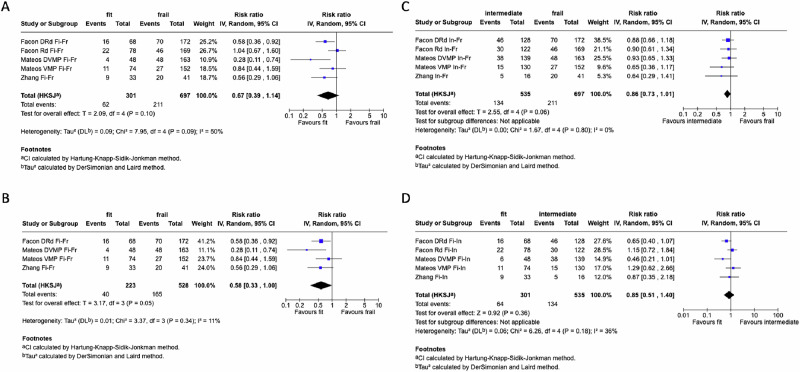
Subgroup analysis for fit versus frail, intermediate versus frail, and fit versus intermediate: meta-analysis. Fi, fit; Fr, frail; In, intermediate; RR, risk ratio. **A** Meta-analysis on fit versus frail subgroup assessing the risk of infection, reported as RR. **B** Meta-analysis on fit versus frail subgroup without the outlier study (Facon Rd Fi/Fr) after leave-on-out sensitivity analysis, assessing the risk of infection, reported as RR. **C** Meta-analysis on intermediate versus frail subgroup assessing the risk of infection, reported as RR. **D** Meta-analysis on fit versus intermediate assessing the risk of infection reported as RR.

A subgroup analysis was conducted to compare infection risk separately for the two categories of non-frail patients *vs* frail ones (Fig. 2). For the subgroup fit *vs* frail, the analysis revealed a RR of 0.67 (95% CI: 0.39–1.14; Fig. 2A; Supplementary Fig. 3A) with *I*^2^ of 50%. In the leave-one-out sensitivity analysis, when excluding the “Facon Rd Fi-Fr” from this subgroup meta-analysis, the RR dropped to 0.58 (95% CI: 0.33–1.00), and *I*^2^ reduced to 0% (Fig. 2B; Supplementary Fig. 3B). For intermediate *vs* frail, the analysis showed a RR of 0.86 (95% CI: 0.73–1.01; *I*^2^ = 0%; Fig. 2C; Supplementary Fig. 3C).

Finally, subgroup analysis was conducted to compare infection risk in fit *vs* intermediate patients (Fig. 2D). The RR was 0.85 (95% CI: 0.51–1.40), with *I*^2^ = 36% (Supplementary Fig. 3D). Notably, in two studies (Facon Rd and Mateos VMP), fit patients paradoxically experienced more infections than intermediate ones. Given the moderate heterogeneity, we conducted a leave-one-out sensitivity analysis, which did not reveal any substantial differences. Then, meta-regression analysis was performed to identify potential sources of heterogeneity among studies. No substantial difference in infection risk was observed, according to study duration, age, proportion of female patients, high cytogenetic risk, neutropenia, lymphocytopenia, leukopenia, thrombocytopenia or anemia (Supplementary Table 4). Nevertheless, the meta-regression showed a significant association between ISS stage III (*p* = 0.007) and the link between frailty and infection risk (Supplementary Fig. 4). This was calculated by comparing the proportion of fit ISS III patients to intermediate ISS III patients within each study. Paradoxically, a lower ISS III ratio, indicating more stage III patients among intermediates, was associated with higher infection risk in the fit group. A separate meta-regression using ISS I stage as a moderator found no significant association (*p* = 0.363), underscoring the inconsistency and limited explanatory value of this relationship.

This meta-analysis confirms that frailty significantly increases infection risk in non-transplant-eligible NDMM patients. The pooled RR for non-frail (fit plus intermediate) versus frail patients was 0.77 (95% CI: 0.65–0.92), indicating a 23% lower risk of severe infections in the more robust group. The lack of heterogeneity (*I*² = 0%) shows this effect was consistent across studies, underscoring that frail MM patients, with impaired immunity and poorer health, face a markedly higher infection risk.

Subgroup analysis examined infection risk across frailty categories. Fit patients showed a lower, though not statistically significant, risk compared to frail patients (RR = 0.67, 95% CI: 0.39–1.14), with moderate heterogeneity (*I*² = 50%) likely due to differences in frailty assessment and treatment regimens. Removing one outlier reduced the RR to 0.58 (95% CI: 0.33–1.00, *p* = 0.05), indicating a 42% lower risk. These findings are consistent with clinical evidence that frail MM patients face higher infection rates due to impaired immunity, disease burden, and treatment-related immunosuppression. The intermediate *vs* frail comparison yielded an RR of 0.86 (95% CI: 0.73–1.01); while fit vs intermediate gave an RR of 0.85 (95% CI: 0.51–1.40) with moderate heterogeneity (*I*² = 36%), indicating some variability among studies. In this latest subgroup analysis, in two studies patients classified as fit experienced higher infection rates than those deemed intermediate, highlighting a paradox that exposes the limitations of current frailty models. Moreover, the meta-regression analysis, used to explain the heterogeneity, showed a significant association with ISS stage III. This finding was inconsistent: studies with more ISS III cases in the intermediate group paradoxically reported higher infection rates among fit patients. Replacing ISS stage III with stage ISS I eliminated the association (*p* = 0.363), casting further doubt on its validity. Overall, disease stage may contribute to variability but is not a reliable predictor of infection risk, which is unsurprising given that ISS was designed to predict survival, not infectious complications. While it may inspire hypotheses, ISS lacks the precision needed for accurate infection-risk stratification.

This meta-analysis has significant clinical implications. Early identification of frail patients enables timely implementation of preventive strategies, including targeted antimicrobial prophylaxis, immunoglobulin replacement in selected cases, and vaccination. Patients classified as intermediate also warrant careful monitoring, as their risk of infection frequently parallels that of frail individuals. On the other hand, two studies reported higher infection rates among fit patients compared with intermediates, underscoring the limited reliability of current frailty classifications, which fail to predict infectious risk with consistency. Because frailty is a dynamic construct, periodic reassessment is essential to guide therapy and to anticipate complications before overt clinical deterioration occurs [14, 15].

However, this study has several limitations, such as the different frailty scores used among studies and the lack of data about immunoglobulins supplementations or antibiotic prophylaxis adopted.

In conclusion, frailty is a key driver of infection risk in non-transplant-eligible patients with MM. However, existing scoring systems lack the precision required for individualized patient care. Frailty assessment should remain central to therapeutic decision-making, but it must be refined to incorporate clinical variables, immune function markers, and functional performance measures. Future research should prioritize the development of improved stratification tools and targeted interventions aimed at reducing infectious complications. Transitioning from a uniform approach to one grounded in precision supportive care, both in clinical trials and in real-world settings, has the potential to markedly improve patient outcomes.

## Supplementary information

Supplementary

## Data Availability

Data will be provided by the author upon reasonable request.
