# Supplementary material for: Impact of frailty on infection risk in non-transplant eligible multiple myeloma patients: a systematic review and meta-analysis
Source: Leukemia. 2026 Feb 17;40(5):1072–5. doi: 10.1038/s41375-026-02880-y (PMC13149299; doi:10.1038/s41375-026-02880-y)
Supplement: Supplementary file 1 — Supplementary [file 41375_2026_2880_MOESM1_ESM.docx]

**Methods. Search strategy and selection criteria.** This systematic review and meta-analysis were performed and reported according to the Preferred Reporting Items for Systematic Reviews and Meta-Analyses (PRISMA) guidelines. The study protocol is registered in PROSPERO (registration ID: CRD420250654904). We conducted a comprehensive search of the MEDLINE and LILACS databases from inception (no backwards time limit) to February 1^st^, 2025, to identify studies evaluating the risk of infections in frailty groups of NDMM patients ineligible for ASCT. The complete list of search terms is detailed in Figure 1 of the appendix. Prospective and retrospective studies were included according to the following inclusion criteria: (1) enrolled non-transplant eligible NDMM patients, (2) classified patients into frailty categories (fit, intermediate, and frail, or non-frail and frail), and (3) reported the number of patients who developed grade 3-4 infections. Studies lacking the necessary data were excluded. No restrictions were applied concerning language or publication date. Additionally, reference lists of included studies, citations, and recent reviews were meticulously screened to identify any further relevant articles.

**Data collection process.** Titles and abstracts were screened, full texts reviewed, data extracted, and the risk of bias or study quality assessed independently by two reviewers (FS and AGS) using a standardized, web-based system ([Rayyan](https://systematicreviewsjournal.biomedcentral.com/articles/10.1186/s13643-016-0384-4)). Any disagreements were resolved through consensus. For each study included, we extracted data on study characteristics, settings, eligibility criteria, populations studied, interventions, and reported outcomes.

**Outcomes.** The risk of infection was the main outcome, and it was calculated as the ratio between the number of patients who developed grade 3-4 infections and the total number of patients within the same frailty group, in each study. Non-frail patients included subjects classified as fit or intermediate (“intermediate” in this meta-analysis). The risk of infection was assessed using the Risk Ratio (RR) as the effect measure. The RR for each study was obtained through meta-analytic pooling, by comparing the proportion of patients who developed grade 3-4 infections in the non-frail groups to the proportion in the frail groups (non-frail vs frail). Moreover, when possible, an additional analysis was performed comparing fit *vs* intermediate patients.

**Data analysis and risk of bias assessment.** Summary measures were pooled using the DerSimonian and Laird random-effects model, with heterogeneity estimated via the Mantel-Haenszel method. Effect size from individual studies were pooled using RR. The summary of findings tables was created through the GRADEpro GDT software (available at [gradepro.org](https://www.gradepro.org/)), and all statistical analyses were performed with ProMeta 3.0 and RevMan software. To assess study quality, we applied the Quality Appraisal of Case Series Studies Checklist developed by the Institute of Health Economics (IHE) (accessible at <http://www.ihe.ca/research-programs/rmd/cssqac/cssqac-about>). Responses were categorized as "yes," "unclear/partial," or "no." Studies were deemed of acceptable quality (low to moderate risk of bias) if ≥70% of responses were "yes". Publication bias was evaluated through funnel plots visual inspection.^14-16^ The quality of evidence was assessed using the GRADE approach. Meta-regression analyses were performed to assess how the magnitude of outcome variables varied based on study-level factors, including: 1) mean age, 2) study duration, 3) proportion of females, 4) International Scoring System stage , 5) high risk cytogenetic and 5) incidence of severe hematologic toxicities (neutropenia, lymphopenia, leukopenia, thrombocytopenia and anemia). Each variable was calculated by comparing the proportion of patients in a specific frailty category to the one in a different frailty category within the same study (e.g., for neutropenia in a single study, the ratio of neutropenic fit patients to neutropenic intermediate patients). To ensure robustness, we also performed a leave-one-out analysis, systematically excluding each study to explore the influence of individual studies on the pooled estimates. Between-study heterogeneity was tested using the 𝝌² test and reported according to the *I²* statistic.

S-Table 1. Quality assessment of the studies.

| Study | **1** | **2** | **3** | **4** | **5** | **6** | **7** | **8** | **9** | **10** | **11** | **12** | **13** | **14** | **15** | **16** | **17** | **18** | **19** | **20** | **Quality score** |
| --- | --- | --- | --- | --- | --- | --- | --- | --- | --- | --- | --- | --- | --- | --- | --- | --- | --- | --- | --- | --- | --- |
| *Mateos*  *2021* | Y | N | Y | Y | Y | Y | Y | Y | Y | Y | Y | Y | Y | Y | Y | Y | Y | Y | Y | Y | 19/20 |
| *Stege*  *2021* | Y | Y | Y | Y | Y | Y | Y | Y | Y | Y | Y | Y | Y | Y | Y | Y | Y | Y | Y | Y | 20/20 |
| *Facon*  *2022* | Y | Y | Y | Y | Y | Y | Y | Y | Y | Y | Y | Y | Y | Y | Y | Y | Y | Y | Y | Y | 20/20 |
| *Groen*  *2023* | Y | Y | Y | Y | Y | Y | Y | Y | Y | Y | Y | Y | Y | Y | Y | Y | Y | Y | Y | Y | 20/20 |
| *Zhang*  *2024* | Y | Y | N | Y | Y | Y | Y | Y | Y | Y | Y | Y | Y | Y | Y | Y | Y | Y | Y | Y | 19/20 |

Y = YES; N = NO; P = PARTIAL; U = UNCLEAR. A study with ≥ 70% of positive responses (12/18 or 14/20) was considered to be of acceptable quality.

Institute of Health Economics (IHE). Quality Appraisal of Case Series Studies Checklist. Edmonton (AB): Institute of Health Economics; 2014. Available from: <http://www.ihe.ca/research-programs/rmd/cssqac/cssqac-about>

References:

Moga C, Guo B, Schopflocher D, Harstall C. Development of a quality appraisal tool for case series studies using a modified Delphi technique. Edmonton: Institute of Health Economics; 2012. Available at <http://www.ihe.ca/advanced-search/development-of-a-quality-appraisal-tool-for-case-series-studies-> [using-a-modified-delphi-technique](http://www.ihe.ca/advanced-search/development-of-a-quality-appraisal-tool-for-case-series-studies-using-a-modified-delphi-technique) . Accessed January 8, 2016.

Guo B, Moga C, Harstall C, Schopflocher D. A principal component analysis is conducted for case series quality appraisal checklist. Journal of Clinical Epidemiology 2016;69:199-207.

S-Table 2. Summary of Findings Table.

| **Certainty assessment** | | | | | | | **№ of patients** | | **Effect** | | **Certainty** | **Importance** |
| --- | --- | --- | --- | --- | --- | --- | --- | --- | --- | --- | --- | --- |
| **№ of studies** | **Study design** | **Risk of bias** | **Inconsistency** | **Indirectness** | **Imprecision** | **Other considerations** | **Non-frail** | **Frail** | **Relative (95% CI)** | **Absolute (95% CI)** |  |  |
| **Infection risk (follow-up: mean 45 weeks; assessed with: mg)** | | | | | | | | | | | | |
| 5^a^ | non-randomised studies and  randomised studies^b^ | not serious^c^ | not serious^d^ | not serious | serious^e^ | all plausible residual confounding would reduce the demonstrated effect dose response gradient^f^ | 901 | 762 | - | RR **0.77**  (0.65 to  0.92) | ⨁⨁⨁⨁  High | CRITICAL |

**CI:** confidence interval; **RR:** risk ratio.

**Explanations**

a. However, Stege et al. and Groen et al. studies are considered a single study for the analysis.

b. The meta-analysis includes randomized and non-randomized studies.

c. All included studies scored above 70% on the IHE checklist, indicating good methodological quality.

d. The I² value is 0%, indicating no heterogeneity. Moreover, the confidence interval of the results is narrow [0.65, 0.92] with a mean effect size of 0.77.

e. All studies cross the threshold of no effect. However, the confidence intervals of the overall effect do not cross the line of no effect. With a statistically significant sample size of at least 314 participants, the meta-analysis provides robust statistical power.

f. Publication Bias: Egger's Test showed Sig=0.137, confirmed by Begg and Mazumdar’s rank correlation test with Sig=0.1 and Trimm and Fill study=0. Then, no significant publication bias has been revealed. Large Effect: the observed effect size (RR = 0.77) suggests a reduced infection risk in non-frail patients compared to frail patients. However, the effect is moderate and does not meet the threshold for a large effect. Plausible confounding: no confounding was found. Dose Response Gradient: a clear dose-response relationship is observed, as infection risk progressively increases from Fit to Intermediate to Frail patients.

S-Table 3. Studies and patients’ characteristics at baseline.

| **study,**  ***year*** | **study**  **type** | **treatment** | **frailty score** | **treatment duration, months, *mean* (cycles)** | **patients at baseline, *n°*** | **age,**  ***mean*** | **female,**  ***n°* (%)** | **ISS**  **Stage,**  ***n°* (%)** | **high cytogenetic risk, n° (%)** | **patients who completed the study, *n°*** | **patients with Grade 3-4 infection,**  ***n°* (%)** |
| --- | --- | --- | --- | --- | --- | --- | --- | --- | --- | --- | --- |
| **Mateos DVMP,**  **2021** | R, MC | DVMP | simplified frailty scale | 30.5 | 350 | 69.7 | 190 (54.3) | I: 69 (19.7)  II: 139 (39.7) III: 142 (56.8) | 53 (15.1) | 346 | 92 (26.6) |
| Fit |  |  |  | 35.9 | 48 | 70 | 25 (52.1) | I: 11 (22.9)  II: 22 (45.8)  III: 15 (31.3) | 7 (15.6) | 48 | 6 (12.5) |
| Intermediate |  |  |  | 35.4 | 139 | 70.6 | 77 (55.4) | I: 39 (28.1)  II: 57 (41.0)  III: 43 (30.9) | 13 (10.4) | 138 | 38 (27.5) |
| Frail |  |  |  | 24.7 | 163 | 73.4 | 88 (54) | I: 19 (11.7)  II: 60 (36.8)  III: 84 (51.5) | 33 (22.9) | 160 | 48 (30) |
| **Mateos VMP,**  **2021** | R, MC | VMP | simplified frailty scale | 11.6 | 356 | 71.2 | 189 (53.1) | I: 67 (18.8)  II: 160 (44.9) III: 129 (36.2) | 45 (12.6) | 354 | 53 (15) |
| Fit |  |  |  | 11.4 | 74 | 70.2 | 34 (45.9) | I: 20 (27.0)  II: 39 (52.7)  III: 15 (20.3) | 9 (14.3) | 74 | 11 (14.9) |
| Intermediate |  |  |  | 11.6 | 130 | 69.6 | 74 (56.9) | I: 24 (18.5)  II: 55 (42.3)  III: 51 (39.2) | 18 (16.7) | 129 | 15 (11.6) |
| Frail |  |  |  | 11.5 | 152 | 73.7 | 81 (53.3) | I: 23 (15.1)  II: 66 (43.4)  III: 63 (41.4) | 18 (13.7) | 151 | 27 (17.9) |
| **Stege,**  **2021** | P, MC | Ixa-Dara-dex | IMWG-FI | 22.7 | 65 | 81 | n.r. | I: 10 (15)  II: 25 (38)  III: 29 (45) | 11 (16.9) | 22 | 16 (25) |
| Frail |  |  |  | 22.7 | 65 | 81 | n.r. | I: 10 (15)  II: 25 (38)  III: 29 (45) | 11 (20) | 22 | 16 (25) |
| **Facon DRd,**  **2022** | P, MC | DRd | simplified frailty scale | 36,4 | 368 | 73.6 | 179 (48.6) | I: 98 (26.6)  II: 163 (44.3)  III: 107 (29.1) | 48 (13) | 225 | 132 (35.9) |
| Fit |  |  |  | / | 68 | 70 | 37 (54.4) | I: 27 (39.7)  II: 27 (39.7)  III: 14 (20.6) | 9 (15.8) | 48 | 16 (23.5) |
| Intermediate |  |  |  | / | 128 | 71.3 | 63 (49.2) | I: 37 (28.9)  II: 62 (48.4)  III: 29 (22.7) | 14 (12.8) | 83 | 46 (35.9) |
| Frail |  |  |  | / | 172 | 76.7 | 79 (45.9) | I: 34 (19.8)  II: 74 (43.0)  III: 64 (37.2) | 25 (16.3) | 94 | 70 (41.7) |
| **Facon Rd,**  **2022** | P, MC | Rd | simplified frailty scale | 36,4 | 369 | 73.6 | 174 (47.2) | I: 103 (27.9)  II: 156 (42.3)  III: 110 (29.8) | 44 (11.9) | 136 | 98 (26.6) |
| Fit |  |  |  | / | 78 | 70.8 | 31 (39.7) | I: 34 (43.6)  II: 31 (39.7)  III: 13 (16.7) | 9 (12.7) | 33 | 22 (28.6) |
| Intermediate |  |  |  | / | 122 | 71.9 | 64 (52.5) | I: 34 (27.9)  II: 58 (47.5)  III: 30 (24.6) | 12 (11.4) | 48 | 30 (24.6) |
| Frail |  |  |  | / | 169 | 76.2 | 79 (46.7) | I: 35 (20.7)  II: 67 (39.6)  III: 67 (39.6) | 23 (15.6) | 55 | 46 (27.7) |
| **Groen,**  **2023** | P, MC | Ixa-Dara-dex | IMWG-FI | 41 | 65 | 75.5 | 30  (46) | I: 16 (25)  II: 37 (57)  III: 12 (18) | 8 (12.3) | 15 | 9 (13.8) |
| Fit-Intermediate |  |  |  | 41 | 65 | 75.5 | 30  (46) | I: 16 (25)  II: 37 (57)  III: 12 (18) | 8 (14) | 15 | 9 (13.8) |
| **Zhang,**  **2024** | P, SC | VRd | DynaFiT | 12 | 90 | 70.7 | 44 (48.9) | I: 7 (7.9)  II: 25 (28.1)  III: 57 (64.0) | 15 (16.7) | 34 | 34 (37.8) |
| Fit |  |  |  | 12 | 33 | 68.5 | 15 (45.5) | I:3 (9.1)  II:15 (45.5)  III:15 (45.5) | 7 (23.3) | 23 | 9 (27.3) |
| Intermediate |  |  |  | 12 | 16 | 69 | 9  (56.3) | I:3 (18.8)  II:3 (18.8)  III:10 (62.5) | 0 (0) | 12 | 5 (31.3) |
| Frail* |  |  |  | 12 | 41 | 72.8 | 20 (48.8) | I:1 (2.5)  II:7 (17.5)  III:32 (80.0) | 8 (24.2) | 21 | 20 (48.8) |
| **Total** |  |  |  | **27.2** | **1,663** | **73.6** | **806 (48.5) ^ω^** | **I: 370 (22.2)**  **II: 705 (42.4)**  **III: 586 (35.2)** | **224 (13.5)** | **1,132** | **434 (26.1)** |

DRd, daratumumab/lenalidomide/dexamethasone; DVMP, daratumumab/bortezomib/melphalan/prednisone; DynaFiT, dynamic frailty-tailored therapy; IMWG-FI, international myeloma working group frailty index; ISS, international staging system; Ixa-Dara-dex, ixatuximab/daratumumab/dexhamethasone; MC, multicenter; P, prospective; R, retrospective; Rd, lenalidomide/ dexamethasone; SC, single center; VCD, bortezomib/cyclophosphamide/dexamethasone; VRd, bortezomib/lenalidomide/dexamethasone; VTD, bortezomib/thalidomide/dexamethasone;

*Daratumumab was administered in 15 frail patients.

**^ω^**The denominator does not include patients that were not assessed for that data.

S-Table 4. Grade 3-4 hematologic toxicities reported across the studies.

| **study,**  ***year*** | **patients at baseline, *n°*** | **neutropenia**  **n°** | **lymphopenia**  **n°** | **leukopenia**  **n°** | **thrombocytopenia**  **n°** | **anemia**  **n°** |
| --- | --- | --- | --- | --- | --- | --- |
| **Mateos DVMP,**  **2021** | 350 | 139 | 26 | 28 | 120 | 60 |
| Fit | 48 | 27 | 6 | 5 | 13 | 5 |
| Intermediate | 139 | 46 | 6 | 10 | 48 | 21 |
| Frail | 163 | 66 | 14 | 13 | 59 | 34 |
| **Mateos VMP,**  **2021** | 356 | 138 | 22 | 12 | 30 | 67 |
| Fit | 74 | 35 | 2 | 4 | 31 | 14 |
| Intermediate | 130 | 51 | 7 | 8 | 44 | 21 |
| Frail | 152 | 52 | 13 | 18 | 59 | 32 |
| **Stege,**  **2021** | 65 | 6 | n.a. | n.a. | 15 | 2 |
| Frail | 65 | 6 | n.a. | n.a. | 15 | 2 |
| **Facon DRd,**  **2022** | 368 | 186 | 56 | 40 | 29 | 49 |
| Fit | 68 | 30 | 7 | 7 | 4 | 4 |
| Intermediate | 128 | 59 | 18 | 11 | 8 | 17 |
| Frail | 172 | 97 | 31 | 22 | 17 | 28 |
| **Facon Rd,**  **2022** | 369 | 129 | 39 | 21 | 33 | 75 |
| Fit | 78 | 22 | 7 | 2 | 3 | 11 |
| Intermediate | 122 | 52 | 14 | 10 | 12 | 24 |
| Frail | 169 | 55 | 18 | 9 | 18 | 40 |
| **Groen,**  **2023** | 65 | 4 | n.a. | n.a. | 3 | 2 |
| Fit-Intermediate | 65 | 4 | n.a. | n.a. | 3 | 2 |
| **Zhang,**  **2024** | 90 | 26 | n.a. | n.a. | 32 | 11 |
| Fit | 33 | 9 | n.a. | n.a. | 10 | 5 |
| Intermediate | 16 | 7 | n.a | n.a. | 8 | 2 |
| Frail | 41 | 9 | n.a. | n.a. | 14 | 4 |

DRd, daratumumab/lenalidomide/dexamethasone; DVMP, daratumumab/bortezomib/melphalan/prednisone; IMWG-FI, international myeloma working group frailty index; ISS, international staging system; Ixa-Dara-dex, ixatuximab/daratumumab/dexhamethasone; MC, multicenter; P, prospective; R, retrospective; Rd, lenalidomide/ dexamethasone; SC, single center; VCD, bortezomib/cyclophosphamide/dexamethasone; VRd, bortezomib/lenalidomide/dexamethasone; VTD, bortezomib/thalidomide/dexamethasone.

S-Figure 1. Flow diagram of research screening.


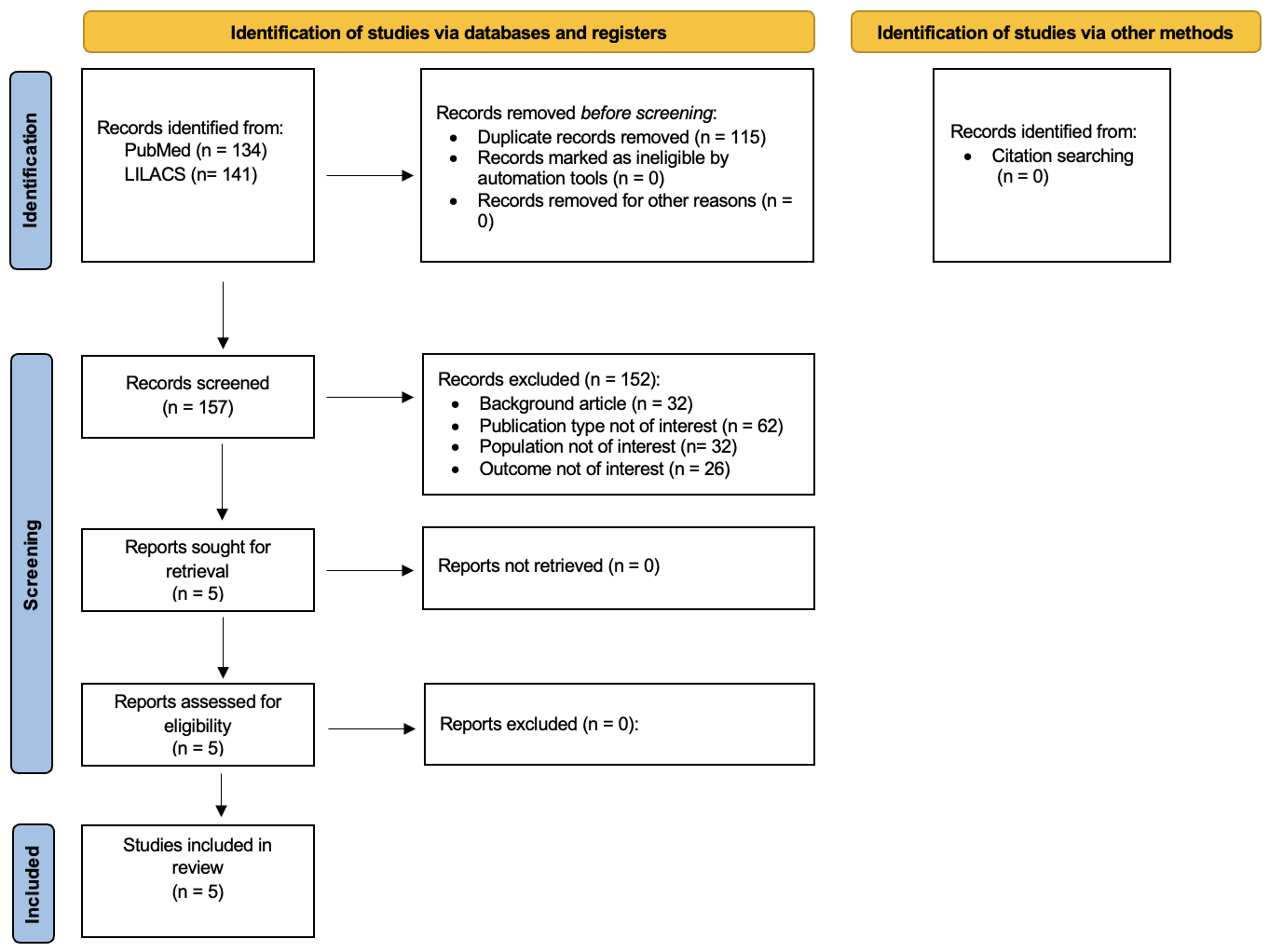


**Medline: (((multiple myeloma[Title]) OR (myeloma[Title])) AND ((frailty[Text Word]) OR (frailty score[Text Word]))) NOT (review[Publication Type])**

#1 Multiple myeloma [Ti] OR myeloma [Ti] - #2 frailty [TW] OR frailty score [TW] - #3 review [PT] - #1 AND #2 NOT #3.

**LILACS: (("multiple myeloma" OR myeloma) AND (frailty OR "frailty score")) AND NOT review**

S-Figure 2. Meta-analysis of prospective studies comparing non-frail versus frail patients.


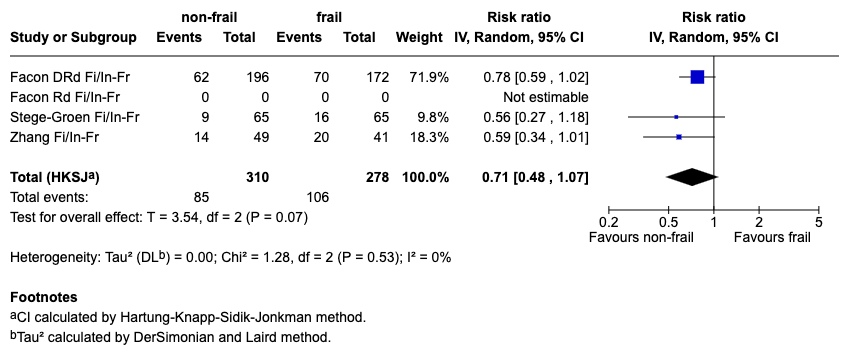


Fi, fit; Fr, frail; In, intermediate; RR, risk ratio.

S-Figure 3. Funnel plots of the subgroups analysis.


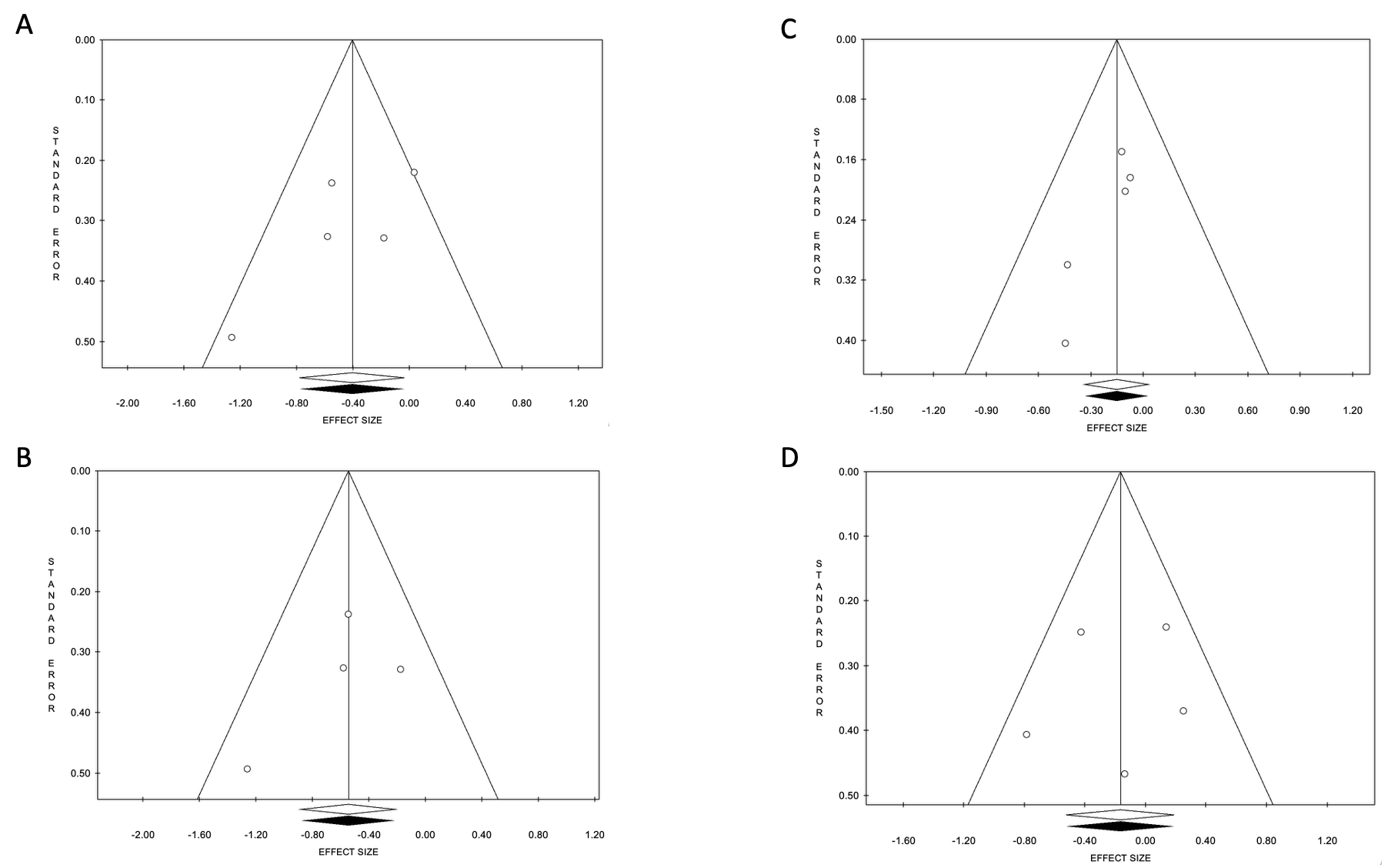


A) fit versus frail funnel plot. B) fit versus frail funnel plot without the outlier study “Facon Rd Fi-Fr”. C) intermediate versus frail funnel plot. D) fit versus intermediate funnel plot.

S-Figure 4. Meta-regression analysis for fit versus intermediate subgroups on infection risk based on the ISS stage III reported in each study.


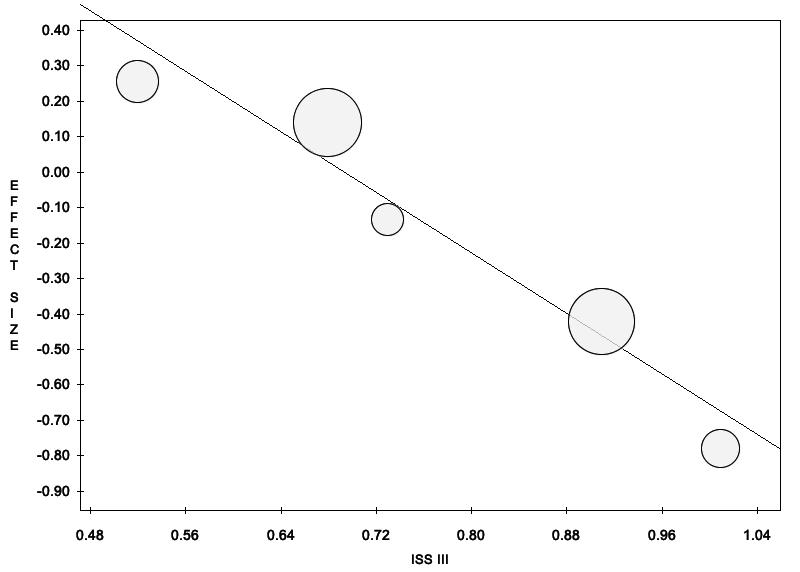


p=0.007

Facon Rd

Mateos VMP

Zhang

Facon DRd

Mateos DVMP

ISS III variable was calculated by comparing the proportion of fit ISS III patients to the proportion of intermediate ISS III within the same study.
